# *N*-GlycositeAtlas: a database resource for mass spectrometry-based human N-linked glycoprotein and glycosylation site mapping

**DOI:** 10.1186/s12014-019-9254-0

**Published:** 2019-09-07

**Authors:** Shisheng Sun, Yingwei Hu, Minghui Ao, Punit Shah, Jing Chen, Weiming Yang, Xingwang Jia, Yuan Tian, Stefani Thomas, Hui Zhang

**Affiliations:** 1 Department of Pathology, Johns Hopkins University, Baltimore, MD 21287 USA; 2 College of Life Science, Northwest University, Xi’an, 710069 Shaanxi China

## Abstract

**Background:**

N-linked glycoprotein is a highly interesting class of proteins for clinical and biological research. The large-scale characterization of N-linked glycoproteins accomplished by mass spectrometry-based glycoproteomics has provided valuable insights into the interdependence of glycoprotein structure and protein function. However, these studies focused mainly on the analysis of specific sample type, and lack the integration of glycoproteomic data from different tissues, body fluids or cell types.

**Methods:**

In this study, we collected the human glycosite-containing peptides identified through their de-glycosylated forms by mass spectrometry from over 100 publications and unpublished datasets generated from our laboratory. A database resource termed *N*-GlycositeAtlas was created and further used for the distribution analyses of glycoproteins among different human cells, tissues and body fluids. Finally, a web interface of *N*-GlycositeAtlas was created to maximize the utility and value of the database.

**Results:**

The *N*-GlycositeAtlas database contains more than 30,000 glycosite-containing peptides (representing > 14,000 N-glycosylation sites) from more than 7200 *N*-glycoproteins from different biological sources including human-derived tissues, body fluids and cell lines from over 100 studies.

**Conclusions:**

The entire human *N*-glycoproteome database as well as 22 sub-databases associated with individual tissues or body fluids can be downloaded from the *N*-GlycositeAtlas website at http://nglycositeatlas.biomarkercenter.org.

## Introduction

It is known that post-translational modifications (PTM) are among the most important factors that increase the diversity of proteins in terms of both structures and functions [1]. The expression analysis of proteins and their PTMs is a key step for the functional characterization of genes and proteins. In the last decade, mass spectrometry has become the most important tool for large-scale proteomic and PTM analysis. Due to the rapid accumulation of a vast amount of proteomic data, many proteome-, sub-proteome-, and protein modification databases have been created in recent years to facilitate proteomic and PTM studies. These databases include ProteomicsDB [2], Human Proteome Map [3], GPMDB [4] and PeptideAtlas [5] for global proteomes; PhosphoSitePlus [6] for phosphorylation sites, acetylation sites, and ubiquitination sites; Unipep [7] for *N*-glycosite-containing peptides; and Cell Surface Protein Atlas [8] for cell surface proteins. The public availability of these databases has facilitated the progress of several studies in their corresponding fields.

Glycosylation is one of the most common PTMs, which plays important roles in many biological processes [9]. Aberrant glycosylation is associated with the pathological progression of many diseases [9]. N-linked glycosylation is a common feature shared by a large fraction of transmembrane proteins, cell surface proteins, and proteins secreted in body fluids [9, 10]. Transmembrane or cell surface glycoproteins are easily accessible to therapeutic drugs, antibodies, and ligands. The glycoproteins secreted in body fluids such as serum, cerebrospinal fluid, and urine are easily accessible and are thought to provide a detailed window into the state of health of an individual. These features make glycoproteins a highly interesting class of proteins for clinical and biological research.

In the last decade, thousands of N-linked glycoproteins have been identified through identifying their glycosite-containing peptides using mass spectrometry [11]. These data have facilitated a better understanding of the glycoprotein contents in humans and other organisms. However, these studies only analyzed specific tissue types, body fluids or cell lines. Unipep is the only database that is specifically dedicated for predicted and identified *N*-glycosite-containing peptides [7], which unfortunately does not contain the information about sources of the identified glycopeptides. Hence, a systematic and integrated analysis of these identified glycoproteins and glycosites is urgently needed.

In this study, we collected more than 30,000 unique human glycosite-containing peptides (de-glycosylated) identified by mass spectrometry, representing > 14,000 unique *N*-glycosites from > 7200 *N*-glycoproteins, from over 100 publications and unpublished datasets. A database resource termed *N*-GlycositeAtlas was created and further used for the distribution analyses of glycoproteins among different human cells, tissues and body fluids. Finally, a web interface of *N*-GlycositeAtlas (http://nglycositeatlas.biomarkercenter.org) was created to maximize the utility and value of the database by providing an online search platform as well as a comprehensive and tissue- or body fluid-specific glycoprotein database that can be downloaded.

## Experimental section

### Collection of N-linked human glycosite-containing peptides

The mass spectrometry identified glycosite-containing peptides from human sources (including tissues, body fluids, and cell lines) were obtained from two main resources: (1) 34 datasets generated from our laboratory (including 15 published and 19 unpublished datasets); (2) 70 papers published by other groups since 2003 (collected on November, 2015). These publications were collected based on their citation of one of the following glycoproteomics technology papers: (1) hydrazide chemistry [12–15]; (2) lectin enrichment [16]; (3) hydrophilic affinity [17]; (4) size extraction chromatography [18]; and (5) FASP-based lectin enrichment [19]. All unpublished glycosite-containing peptides were enriched using the hydrazide chemistry (SPEG) method [12, 13] from different human-related samples. It should be noted that only glycosite-containing peptides identified by their de-glycosylated forms were collected, the glycoproteins identified through intact glycopeptides or other non-glycosylated peptides were not included in this study. After glycosite-containing peptide collection from these published papers, the data were further filtered by N-X-S/T motif (X can be any amino acid except proline) with deamidation (de-glycosylated form) at the asparagine residue. In order to keep the original records from published papers, no further quality control step was performed prior to the database assembly.

Among these unpublished datasets generated in our laboratory, eleven of them were generated before 2008 and have been included in the Unipep website (http://www.unipep.org) [7] and/or PeptideAtlas website (http://www.peptideatlas.org) [20]. These samples were enriched by the SPEG method and analyzed by an LTQ ion trap (Thermo Fisher, San Jose, CA) or Q-TOF (Waters, Beverly, MA) mass spectrometers followed by being searched with the SEQUEST algorithm [21] against a human International Protein Index database (IPI) [22]. The peptide mass tolerance was 2.0 Da. Carbamidomethylation (C, + 57.0215 Da) was set as a static modification; oxidation (M, + 15.9949 Da) and deamination (N, + 0.98 Da) were set as dynamic modifications. The output files were further evaluated by INTERACT and ProteinProphet [23, 24]. The identified peptides were filtered by a PeptideProphet probability score ≥ 0.9 and the deamidation of asparagine (N) in the N-X-S/T motif. The identification of glycosite-containing peptides from these data was filtered by deamidation (de-glycosylated form) in the N-X-S/T motif.

The other eight big datasets were generated using Orbitrap Velos and/or Q-Exactive mass spectrometers (Thermo Fisher Scientific, Bremen, Germany) after former glycopeptide enrichment using SPEG method and searched against an NCBI Reference Sequence (RefSeq) human protein database [25] using SEQUEST [21] in Proteome Discoverer v1.4 (Thermo Fisher Scientific). The database searching parameters for glycosite-containing peptide identification were set as follows: two missed cleavages were allowed for trypsin digestion with 10 ppm precursor mass tolerance and 0.06 Da fragment mass tolerance. Carbamidomethylation (C) was set as a static modification, while oxidation (M) and deamination (N) were set as dynamic modifications. For iTRAQ-labeled samples, iTRAQ-4plex (peptide *N*-terminal) and iTRAQ-4plex (K) were added as dynamic modifications. The glycosite-containing peptide identifications were filtered by 1% FDR and deamination in the N-X-S/T motif of the peptides. Four of these unpublished datasets (raw data) have been deposited to the ProteomeXchange Consortium (http://proteomecentral.proteomexchange.org) via the PRIDE partner repository [26] with the dataset identifier PXD005143. Another glycoproteome dataset is accessible through the Clinical Proteomic Tumor Analysis Consortium (CPTAC) website (https://cptac-data-portal.georgetown.edu/cptac/s/S020).

### Glycoprotein mapping and database assembly

All identified glycosite-containing peptides from different published papers and unpublished datasets were matched to the UniProt human protein database (downloaded at Nov. 3rd, 2015 from website http://www.uniprot.org) using an in-house software. Using this in-house software, all glycosite-containing peptides were first mapped into the reviewed UniProt database, and unmatched peptides were further mapped into an un-reviewed UniProt database. The matched protein IDs, gene names, protein names, glycosylation site locations, and peptide sequences with ± 20 amino acids surrounding each glycosite (N-X-S/T motif, X ≠ P) were extracted and assembled into a human glycoprotein and glycosite database, termed *N*-GlycositeAtlas. When a peptide could match to more than one protein, all protein records were included in the database. In addition, only peptides containing the typical N-X-S/T N-glycosylation motif were included in the database.

### Data access

The *N*-GlycositeAtlas is accessible at http://nglycositeatlas.biomarkercenter.org. The user can download the entire and 22 tissue/body fluid specific human glycoprotein databases from the website.

## Results and discussions

### Assembly of *N*-GlycositeAtlas

**Fig. 1:**
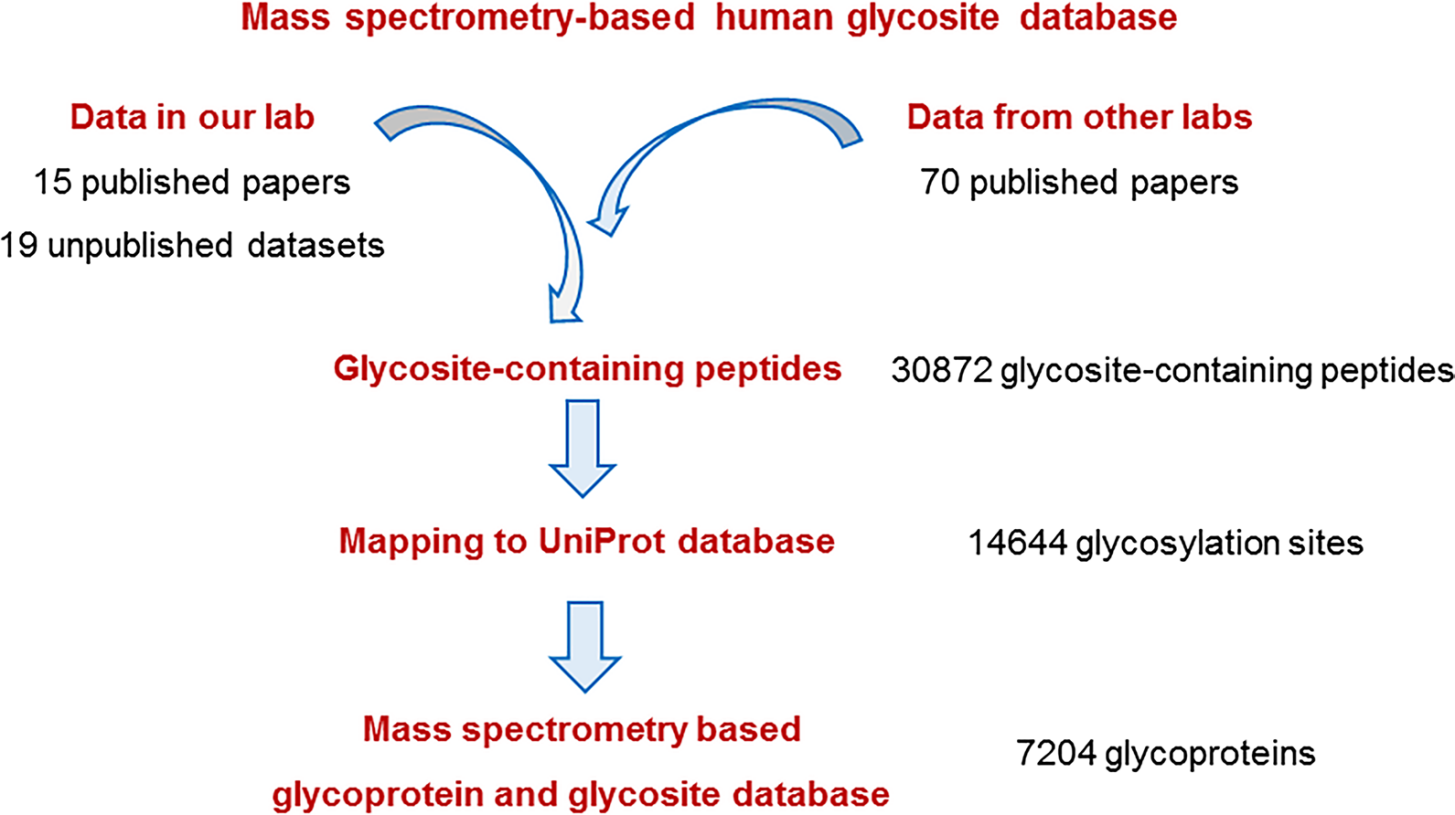
Assembly of the mass spectrometry-based human glycoprotein and glycosite database (*N*-GlycositeAtlas). The identified glycosite-containing peptides were collected from 85 publications and 19 newly generated or unpublished datasets. The peptides were then matched to a UniProt protein database. The relevant information for each glycosite was then extracted for glycosite and glycoprotein database development

Here, we present a mass spectrometry-identified N-linked glycoprotein and glycosite database, named *N*-GlycositeAtlas, to facilitate human protein glycosylation studies. The human glycosite-containing peptides identified via their de-glycosylated forms were initially collected from all human glycosylation-related datasets with thousands of LC–MS/MS data including 15 published [7, 27–40] and 19 newly generated datasets (> 1000 LC–MS/MS files) generated in our laboratory (Fig. 1). The glycosite-containing peptides were then matched to their proteins in a common UniProt human protein database (http://www.uniprot.org). For each matched protein, the protein accession number, protein name, gene name, N-linked glycosylation location and the protein sequence at ± 20 amino acids surrounding the glycosylation site were extracted from the protein database to constitute the *N*-GlycositeAtlas. Using this strategy, we collected 13,811 human glycosite-containing peptides representing 11,336 unique glycosites from 34 datasets generated in our laboratory (Fig. 1).

To expand the database, we also collected human glycosite-containing peptides from all papers regarding to human glycosite-containing peptide analysis published since 2003. Using the same strategy as above, we eventually collected 22,618 glycosite-containing peptides that belong to 8818 unique glycosites from 70 papers published by other laboratories [7, 14, 15, 17, 18, 33, 41–104]. Altogether, the *N*-GlycositeAtlas contains 30,872 unique glycosite-containing peptides that match to 14,644 unique glycosites in 7204 glycoproteins (Fig. 1 and Additional file 1: Table S1).

**Fig. 2:**
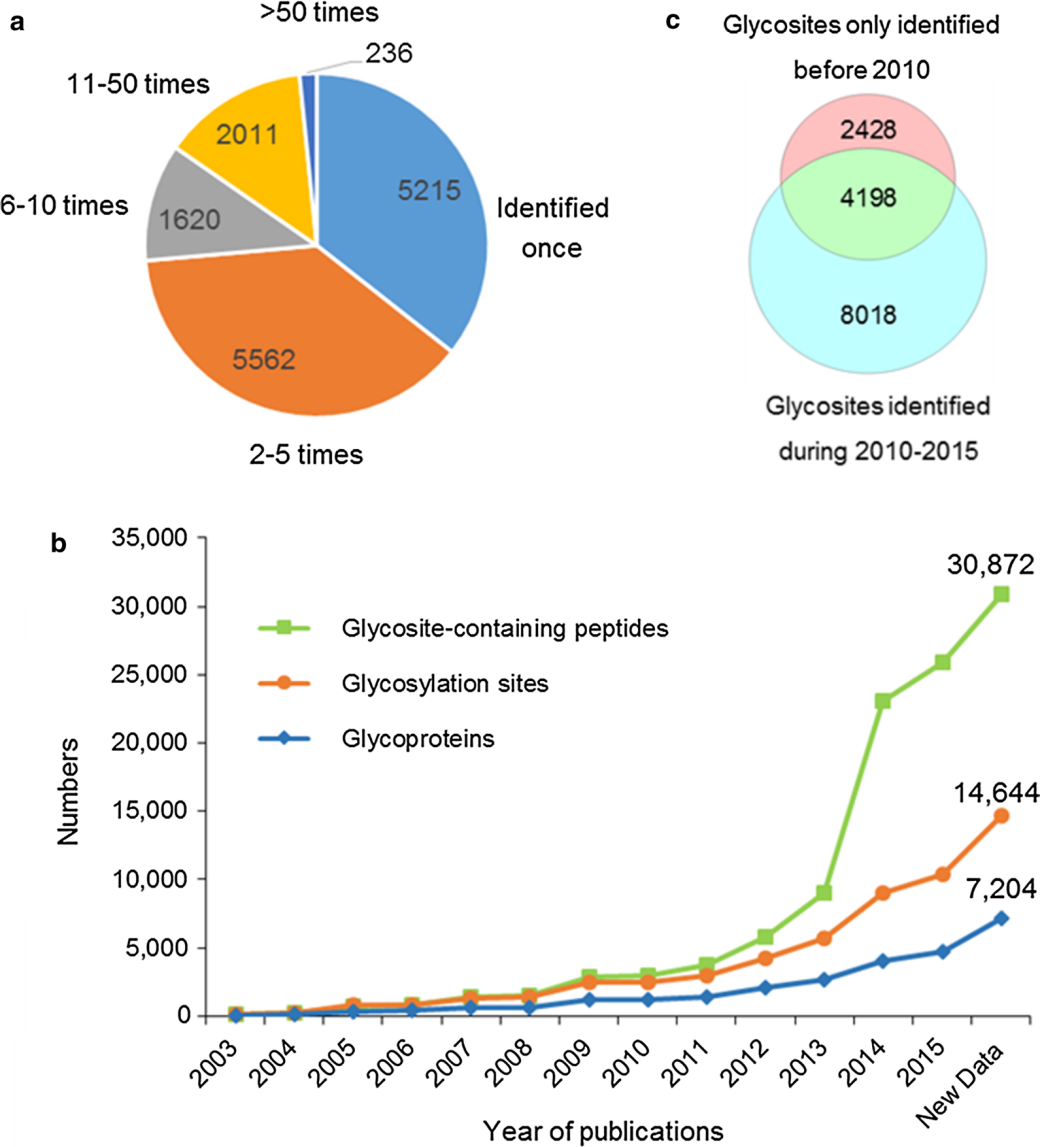
Overview of the human glycoprotein and glycosite database. **a** The identification frequencies of each glycosite in the database. The identification frequencies of each glycosite were determined based on their identification in different samples, different studies or glycosite-containing peptides of various lengths (due to different enzyme digestion or different missed cleavages). **b** Accumulation of identified glycosite-containing peptides, unique glycosites and glycoproteins with time. **c** Classification of glycosites according to their year of publication

The confidence of the data is one of most important considerations in measuring the quality of a database. In *N*-GlycositeAtlas, only peptides containing the typical N-X-S/T N-glycosylation motif were included in the database to ensure the high confidence of the data, even though recent studies indicated that *N*-glycans can also be attached to other atypical motifs [19, 40]. In addition, all glycosite-containing peptides identified from studies conducted in our laboratory must contain ≥ 1 deamidation site at the former glycosylation sites (after PNGase F treatment). It is considerably more difficult for us to control the quality of the data published from other groups. Nevertheless, the confidence of a given glycosite or glycosite-containing peptide could still be estimated according to its identification frequency. Generally, glycosites that were identified more frequently from different samples or studies (based on either the same glycosite-containing peptide or a glycosite-containing peptide with a different length resulting from missed cleavages or different enzyme digestion) had a higher confidence. In *N*-GlycositeAtlas, 2247 glycosites were identified more than 10 times, 7182 glycosites were identified 2-10 times, and 5215 glycosites (35.6%) were identified only once in all different datasets (Fig. 2a).

The confidence of these identified glycosites, especially the glycosites that were only identified once, can be further estimated based on their original mass spectrometry data and subsequent analytical methods. Owing to the huge improvement of mass spectrometry technology, liquid chromatography (LC) separation, analysis software and glycoprotein/glycosite-containing peptide isolation methods in recent years, the number of confidently identified N-linked glycosite-containing peptides has increased dramatically. As most of the glycosite-containing peptides in *N*-GlycositeAtlas were identified as their de-glycosylation form with deamidation (+ 0.98 Da) at the former glycosites (after PNGase F treatment), the high resolution and accuracy of the mass spectrometers that were used to conduct these studies in recent years greatly increased the identification confidence of the glycosites and glycoproteins as well as increased the numbers of identified glycosite-containing peptides at pre-determined false discovery rates (FDR). In order to estimate the confidence of the glycosite-containing peptides in the database using this information, we simply analyzed the data according to their date of publication. Our results showed that although the identified human glycoproteins and glycosites have been steadily increasing since 2003 when the first two glycoproteomic studies were published [12, 16], the huge increase mainly occurred in recent years (Fig. 2b). We found that the majority of the glycosites (83.4%) in the database were published during 2010–2015 (Fig. 2c), and these sites were most likely identified with high confidence by using high resolution and high accurate mass spectrometry.

Additional information about the detailed mass spectrometers and search parameters for the identification of a given glycosite or glycoprotein can be obtained from the original publications listed in the database.

### Distribution of glycoproteins and glycosites across tissues and biological fluids

**Fig. 3:**
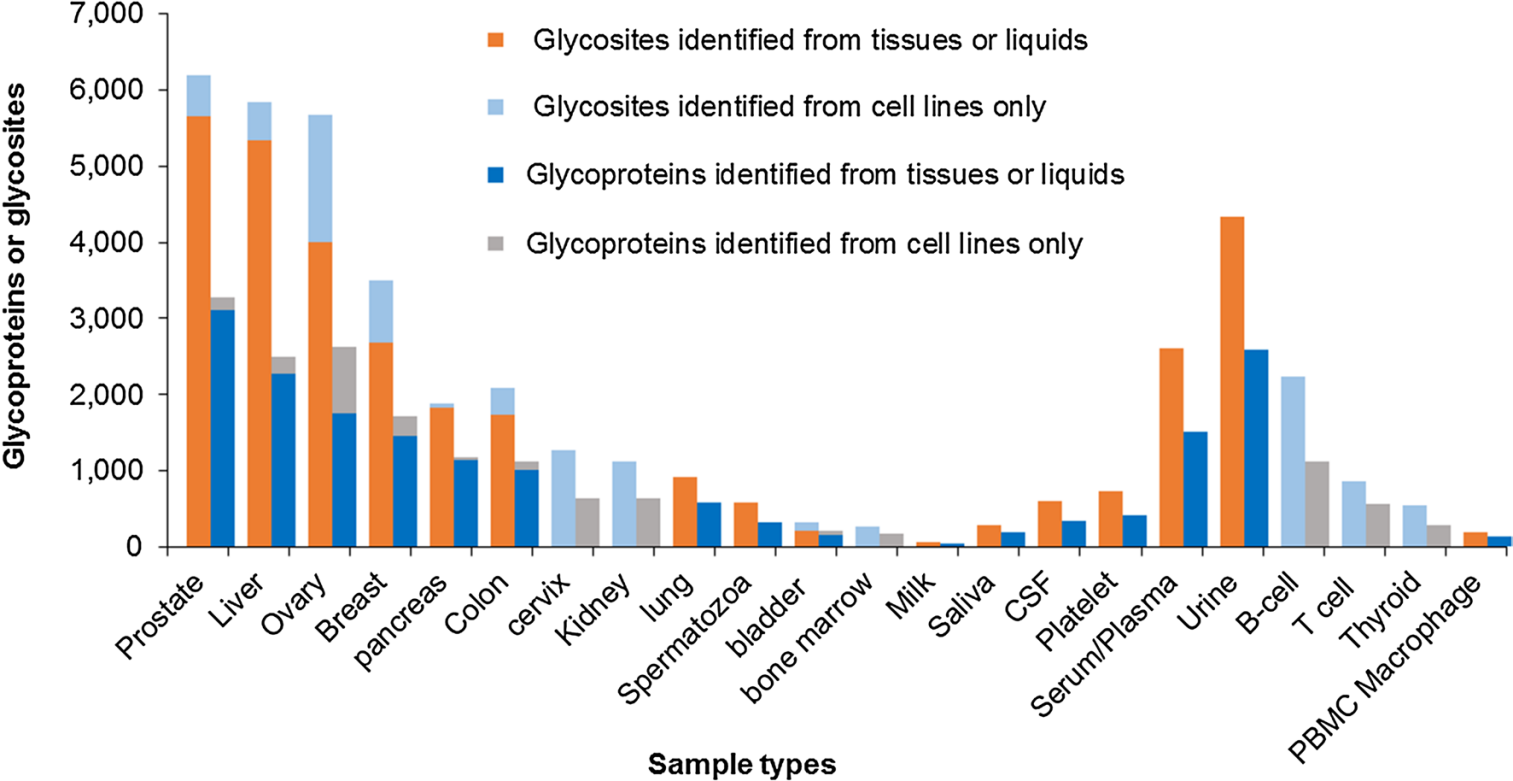
Distribution of identified glycosites and glycoproteins across different human tissues or body fluids. The blue columns represent the glycosites (**a**) and glycoproteins (**b**) identified from human tissues or body fluids; the orange columns represent the glycosites (**a**) and glycoproteins (**b**) identified from the related cell lines. *CSF* cerebrospinal fluid, *PBMC* peripheral blood mononuclear cell

Determining the current status of glycoprotein analysis in each human tissue and body fluid will benefit future human glycoproteomic studies. Using *N*-GlycositeAtlas, we investigated the distribution of identified glycoproteins across different human tissues and body fluids. Among eight tissues including prostate, liver, ovary, breast, pancreas, colon, lung and bladder, prostate has the most number of identified glycosites (> 6000; Fig. 3a) and glycoproteins (> 3000; Fig. 3b). There were also more than 5000 glycosites and 2000 glycoproteins identified from liver and ovary. In addition to the tissue glycoproteins, 311 glycoproteins with 585 glycosites were identified from spermatozoa [71]. However, there are still many tissues with no glycoproteomics data or with only limited data obtained from the related cell lines. In fact, this is the case even with many essential organs of the human body, such as heart, stomach, brain, and kidney. Glycoproteomic analysis of these tissues will promote human glycoproteomics studies and enhance our understanding of the distribution and function of glycoproteins in different tissues.

As N-linked glycoproteins account for a large portion of the protein content in serum and other body fluids, identifying the glycoprotein components in these body fluids is essential for their clinical utility. *N*-GlycositeAtlas contains 2645 and 1845 glycoproteins that were identified from urine and serum, respectively (Fig. 3). Based on these results, we found that more glycoproteins were identified from urine than from serum. The possible reason is that serum contains many high abundant glycoproteins, and these glycoproteins might inhibit the identification of low abundant glycoproteins in serum. Removal of these high abundant proteins before mass spectrometry-based proteomic or sub-proteomic analyses would increase the number of identified serum glycoproteins [14]. Several hundred glycoproteins have also been identified from saliva and cerebrospinal fluid (CSF). In addition, > 1000 glycoproteins have been detected from platelets and T cell cell lines, and > 500 glycoproteins have been identified from B-cell cell lines (Fig. 3).

The glycoprotein and glycosite databases associated with individual tissues or body fluids can be downloaded from the *N*-GlycositeAtlas website.

### Comparison of serum and urinary glycoproteins with tissue-derived glycoproteins

**Fig. 4:**
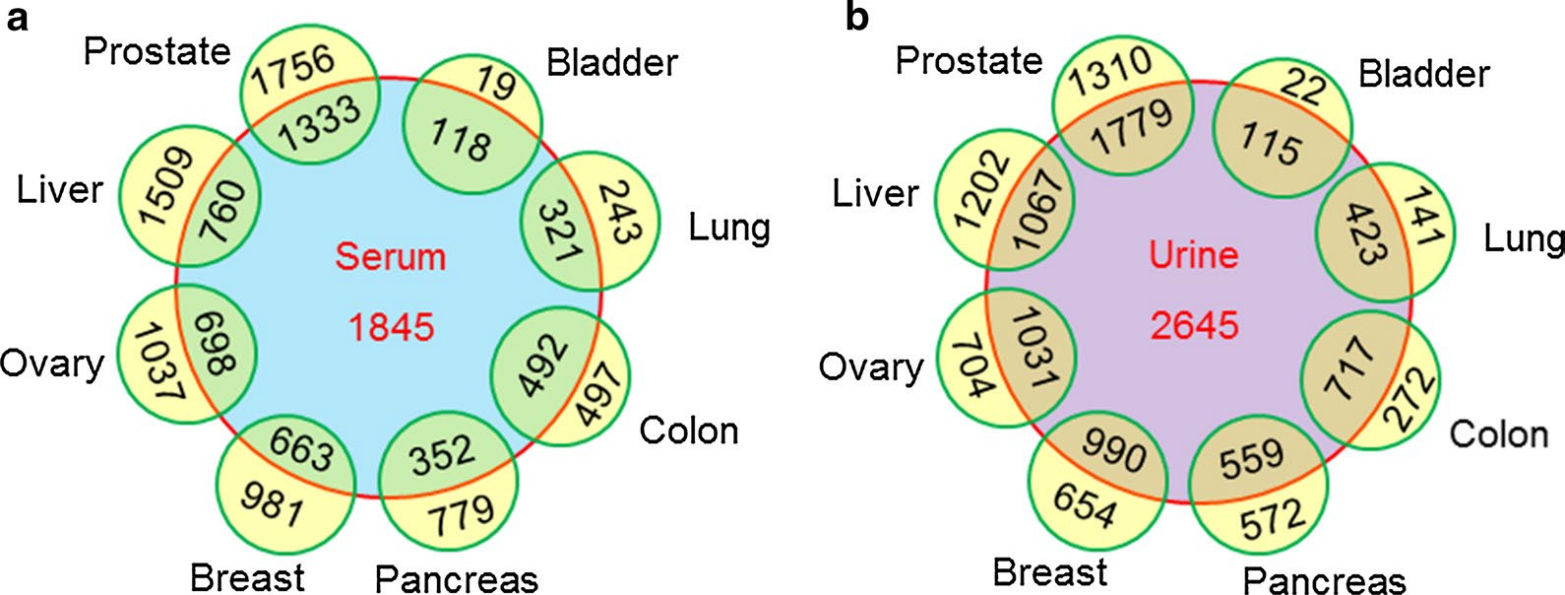
Glycoproteins identified in common between tissues and serum (**a**) or urine (**b**)

Serum is the most widely used biospecimen for disease detection and monitoring due to its ease of access and rich physiological and pathological information. The detection of disease-related glycoprotein changes in serum is an important strategy for disease biomarker discovery [105]. Using the data in *N*-GlycositeAtlas, we compared the glycoprotein contents between serum and eight different tissues (cell line-related glycoproteins were not included) to investigate the detectability of tissue glycoproteins in serum. The results indicated that different tissues had different numbers and percentages of glycoproteins overlapped with serum-derived glycoproteins (Fig. 4a). An average of 47.6 ± 16.5% glycoproteins identified in tissues were also detected in serum. The data confirmed the high value of serum tests in the detection of glycoprotein changes associated with various diseases.

Body fluids other than serum such as urine and CSF are also important specimens for clinical tests. In this study, we also analyzed urine-derived glycoproteins based on the clinical utility of urine. The urinary glycoproteins were also compared with glycoproteins from eight different tissues. The results indicated that a lot of glycoproteins were also commonly identified from urine and tissues, with an average of 63.1 ± 12.1% tissue-derived glycoproteins overlapping with urine-derived glycoproteins (Fig. 4b). More tissue-derived glycoproteins were detected in urine than in serum, which could be attributed to the larger number of glycoproteins that were identified in urine compared to serum. To further investigate the potential of urine in clinical tests and biomarker discovery, we also compared the glycoproteins between urine and serum. Among 1845 glycoproteins identified in serum, 827 (44.8%) were also identified in urine. The abundance glycoprotein content in urine and the high percentage of glycoproteins that overlap with tissue-derived glycoproteins suggests the high potential of urine in clinical detection and biomarker discovery. However, additional studies are required to confirm whether these urinary glycoproteins change with disease and reflect different pathological states within different parts of the human body.

### *N*-GlycositeAtlas web interface

**Fig. 5:**
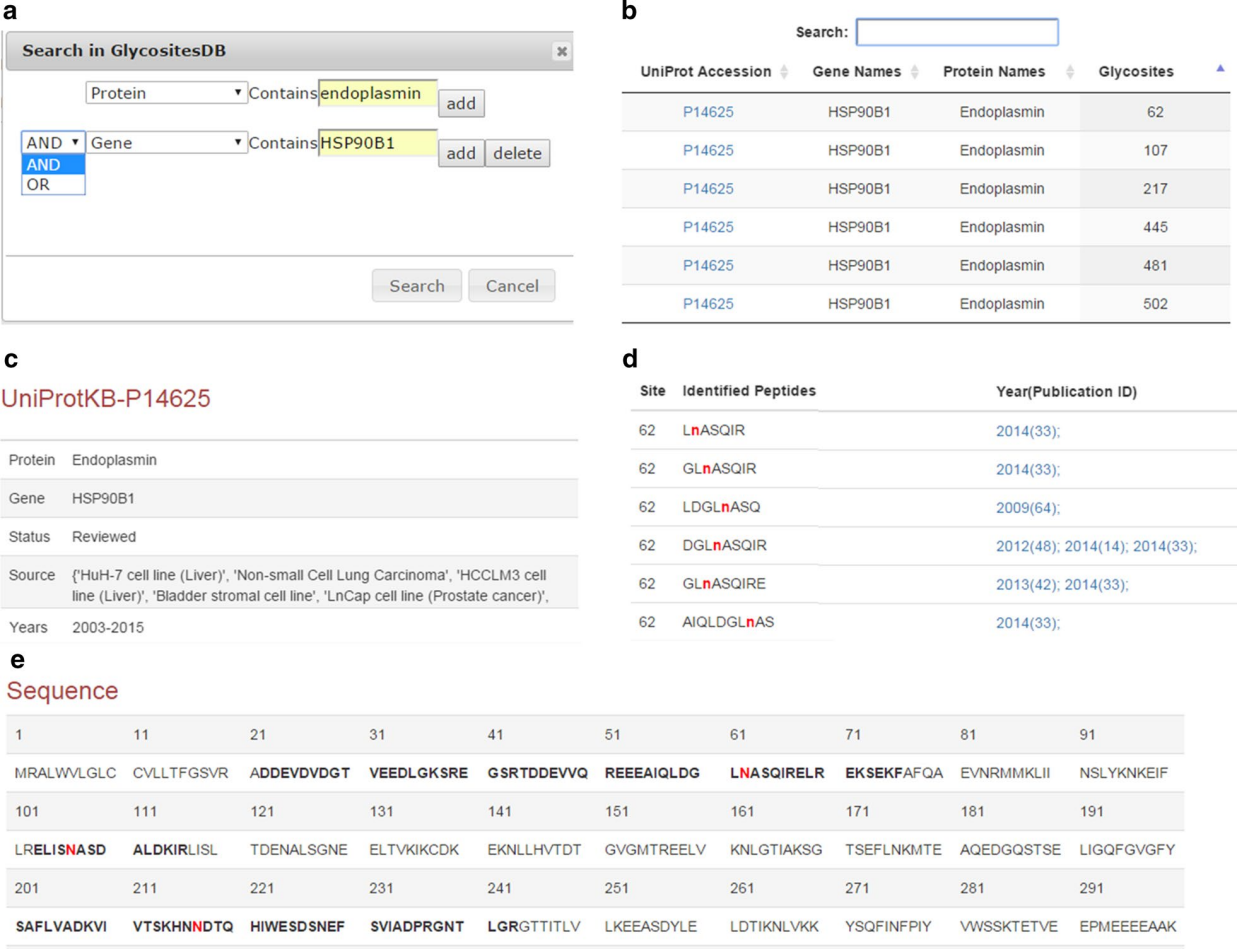
Representative *N*-GlycositeAtlas web interface output showing an N-linked glycoprotein and its glycosites. Endoplasmin (HSP90B1) is used as an example. **a** The database search. The database can be searched online according to the protein accession number, gene name, protein name, glycosylation site location, glycosite-containing peptide, N-glycosylation motif (N-X-S/T), name of tissue/liquid/cell line, year of publication, and/or reference. Multiple search parameters can be used for a combined or parameter-specific search. **b** The search results are shown in the first display page. In the first display page, the glycoprotein accession number (UniProt), gene name, protein name and glycosylation site location are exhibited. The detailed information for each glycoprotein can be accessed in the second display page by clicking the glycoprotein accession number. In the second display page, the following information is shown: **c** glycoprotein information; **d** glycosite and glycosite-containing peptide information of the glycoprotein as well as their references; and **e** glycosites (red) and glycosite-containing peptides (bold font) highlighted in the protein sequence

To make the database readily accessible and easy to update, we designed a web interface (http://nglycositeatlas.biomarkercenter.org) to facilitate the online searching of the database and the downloading of data. By using the web interface, users can easily search the database either using the general search function for basic search or advanced search by restricting the search based on protein accession number, gene name, protein name, glycosylation site location, glycosite-containing peptide, N-glycosylation motif (N-X-S/T), the name of tissue/liquid/cell line, year of publication, and/or reference for specific searches (Fig. 5a). Multiple search parameters can be used together for multiple searches (link with “or”) or more specific searches (link with “and”, Fig. 5a).

We designed two layers of display pages to exhibit the results. The first layer of the display page only exhibits general information of the glycoproteins, including glycoprotein accession numbers (UniProt), gene names, protein names and identified glycosylation sites (Fig. 5b). The additional information for each glycoprotein can be gained in the second display page by clicking the related glycoprotein accession number. In the second display page, the user will obtain the tissue/liquid/cell line types where the glycoprotein was identified (Fig. 5c), all glycosite-containing peptides identified at each glycosite with the reference information (Fig. 5d), as well as the highlighted the location of the identified glycosites and glycosite-containing peptides in the protein sequence (Fig. 5e).

In addition, the entire human glycoprotein and glycosite database as well as the glycoprotein database for each individual tissue or body fluid can also be downloaded from the *N*-GlycositeAtlas website in a Microsoft Excel format. The following information is included in the database: (1) UniProt accession numbers of glycoproteins; (2) whether the protein has been reviewed in the UniProt database; (3) protein names; (4) gene names; (5) location of the glycosylation sites; (6) identified glycosite-containing peptides; (7) the protein sequence at ± 20 amino acids surrounding the identified glycosylation site; (8) names of tissues/body fluids/cell lines where the glycosite-containing peptide was identified; (9) year of publication; and (10) references. It should be noted that each line of text only contains one glycosite-containing peptide and one glycosite location. When a peptide contains more than one glycosite, each glycosite is displayed on a separate line. In addition, different proteins are also listed on separate lines when one glycosite-containing peptide was matched to more than one protein. The detailed information for each identified glycosite or glycosite-containing protein can be acquired from their original publications that are listed after each record.

## Conclusions

In this study, we created a human glycoprotein and glycosite database containing > 14,000 *N*-glycosites and more than 7200 *N*-glycoproteins that were identified through their de-glycosylated forms of glycosite-containing peptides by mass spectrometry from over 100 publications or unpublished datasets. Based on the data in the database, we observed that although several thousand glycoproteins could be identified from one single tissue, there were still many tissues where no mass spectrometry-based glycosite data has been generated yet. A considerable amount of additional work is still needed to profile the human glycoproteomes at the human genomic level. Many common glycoproteins identified between tissues and serum confirmed the high value of serum in clinical tests, while the large proportion of common glycoproteins between different tissues and urine suggested the high potential of urine for clinical detection and biomarker discovery. Finally, the web interface of *N*-GlycositeAtlas (http://nglycositeatlas.biomarkercenter.org) was created to maximize the utility and value of the database by providing an online search platform as well as a comprehensive and tissue- or body fluid-specific glycoprotein database that can be downloaded.

## Supplementary information

**Additional file 1: Table S1.** The mass spectrometry-based human glycoprotein and glycosite database.

## Abbreviations

Glycosite: N-linked glycosylation site
LC–MS/MS: liquid chromatography combined with tandem-mass spectrometry
CSF: cerebrospinal fluid
PBMC: peripheral blood mononuclear cell
